# The Economic Burden of Colorectal Cancer in Type 2 Diabetes Mellitus (T2DM) in Shanghai, China

**DOI:** 10.1002/cam4.70651

**Published:** 2025-02-21

**Authors:** Zhi‐xu Zhu, Yu‐xin Peng, Jing‐hong Ye, Yan Wei

**Affiliations:** ^1^ School of Public Health Fudan University Shanghai People's Republic of China; ^2^ NHC Key Laboratory of Health Technology Assessment Fudan University Shanghai People's Republic of China; ^3^ Hongkou District Center for Disease Control and Prevention, Shanghai Hongkou District Shanghai People's Republic of China

**Keywords:** colorectal cancer, economic burden, propensity score matching, type 2 diabetes mellitus

## Abstract

**Objective:**

This study aimed to analyze disease cost levels among colorectal cancer (CRC) patients, examining differences in disease costs between those with type 2 diabetes mellitus (T2DM) and those without T2DM while considering various demographic characteristics.

**Methods:**

The study included respondents from colorectal cancer patients diagnosed and managed within the population‐based cancer registry system of the Shanghai Municipal Center for Disease Control and Prevention from 2020 to 2022. Diabetic colorectal cancer cases were matched with nondiabetic colorectal cancer controls using propensity scores, maintaining a 1:1 ratio between cases and controls; bias for the majority of the matched variables is below 10%. Direct, indirect, and intangible costs were estimated for study subjects.

**Results:**

After propensity score matching, the analysis encompassed 376 cases of T2DM‐related CRC and 376 non‐T2DM CRC cases. The T2DM group exhibited higher direct medical costs (¥57,059.65 vs. ¥48,933.93, *p* < 0.05), direct nonmedical costs (¥9292.45 vs. ¥7969.35), indirect costs (¥300.13 vs. ¥241.11), intangible costs (¥3601.70 vs. ¥2631.96), and total disease costs (¥70,253.93 vs. ¥59,776.36, *p* < 0.05) compared to the non‐T2DM group. In Stage II CRC, direct medical costs were ¥74,008.39 for the T2DM group versus ¥57,368.84 for the non‐T2DM group. Among surgical patients, direct medical costs were ¥57,658.81 for the T2DM group versus ¥1337.00 for the non‐T2DM group, and ¥49,061.52 for the non‐T2DM surgical group versus ¥1089.00 for the non‐T2DM nonsurgical group.

**Conclusion:**

Colorectal cancer imposes a substantial economic burden. Patients with CRC and concurrent T2DM experience a higher economic burden compared to nondiabetic patients. Notably, individuals with both T2DM and Stage II cancer face a significantly higher economic burden, whereas surgical patients exhibit a significantly greater disease burden than nonsurgical patients. Efforts should concentrate on primary prevention and secondary prevention to alleviate the economic burden associated with colorectal cancer.

## 1 Introduction

Globally, colorectal cancer (CRC) stands as the third most prevalent cancer, following breast and lung cancer, with nearly two million new diagnoses in 2020 alone [1]. In China, the latest statistical report from the National Cancer Center (NCC) indicates that the incidence rate of CRC is 36.63 per 100,000, respectively, making CRC the second most common cancer in terms of new cases [2]. Notably, its incidence is increasing across both younger and older demographics. Although an aging population partially explains this surge, the escalating rates among younger patients remain a subject of ongoing investigation [3, 4]. Remarkable progress in CRC treatment outcomes over recent decades can be attributed to a deeper understanding of its molecular mechanisms, enhanced early detection methods, and advancements in both systemic and locoregional therapies. Some studies now report 5‐year survival rates for CRC as high as 64% [5]. Despite these advancements, the financial implications of CRC treatment are considerable, exerting a substantial burden on governments, healthcare systems, and affected families.

The burden of CRC correlates strongly with socioeconomic factors, exhibiting significant geographic variations [6]. Countries with higher human development indices often face increased CRC incidence and mortality, with Europe, Oceania, and Northern America witnessing the highest rates [3]. In recent years, shifts in economic conditions, lifestyle, and dietary habits have contributed to a rise in CRC incidence and mortality in China [1, 7]. In 2020, new CRC cases in China represented 28.8% of the global total, with CRC‐associated deaths accounting for 30.6% of worldwide CRC fatalities [1], thereby placing a significant strain on the healthcare system.

Diabetes mellitus has been identified as a risk factor for CRC, with epidemiological studies highlighting its association with increased CRC risk [8]. A recent study published in JAMA found that the proportion of CRC patients with diabetes in the United States is 1.1% [9]. The results indicate that diabetes is associated with an increased risk of CRC (HR = 1.47). Potential pathophysiological mechanisms include hyperinsulinemia, the insulin‐like growth factor (IGF) axis, hyperglycemia, inflammation due to adipose tissue dysfunction, gastrointestinal motility disorders, and impaired immunological surveillance. Studies indicate that diabetes can adversely affect CRC prognosis [10], suggesting that pharmacological or lifestyle interventions aimed at reducing insulin levels may be effective in preventing CRC tumorigenesis [11]. These factors potentially contribute to variations in the cost of disease among CRC patients with diabetes.

This study addresses a gap in the literature concerning the economic impact of CRC in the Chinese population, particularly considering the cost implications of diabetes as a complicating factor. Although most existing research has focused on direct and indirect costs of CRC, the influence of diabetes‐related complications on disease costs has been less explored. Our objective is to ascertain whether there is a notable difference in the cost of disease between diabetic and nondiabetic CRC patients, based on an analysis of disease cost levels.

However, our findings may be subject to residual confounding due to variations in baseline participant characteristics. To mitigate this, we have employed propensity score matching (PSM) to ensure comparability of baseline characteristics between diabetic and nondiabetic CRC patients. PSM is a robust method for balancing covariate distributions across groups, thus enhancing the validity of our conclusions [12].

## 2 Materials and Methods

### 2.1 Study Population and Design

This study centered on CRC patients registered in the Shanghai Municipal Center for Disease Control and Prevention's population‐based cancer registry between 2020 and 2022. Utilizing a nested case–control approach, nondiabetic CRC patients were methodically matched with diabetic counterparts. These matches were based on age, year of diagnosis, and community‐based management to ensure comparability between groups.

A meticulously designed questionnaire, developed through extensive expert consultation and preliminary testing, covered two critical areas: the economic burden of CRC and patient quality of life. This comprehensive electronic questionnaire was subsequently administered to CRC patients who were under the management of family physicians in community health service centers. The survey period spanned from November 1, 2022, to January 3, 2023.

### 2.2 Participants With Type 2 Diabetes Mellitus (T2DM)

Participants included CRC patients from a cohort study on T2DM‐related complications and mortality in Shanghai's Hongkou District, initiated in 2018 with a total inclusion of 20,251 patients. The inclusion criteria for our study were specifically focused on CRC patients diagnosed within the 2020–2022 timeframe. These patients were identified based on their CRC diagnosis (ICD‐10 C18‐C20) in secondary and tertiary hospitals, their residence in Hongkou District, and their engagement with community health service center management. Exclusion criteria were applied to those who had relocated, refused participation, or passed away. In total, 383 patients with T2DM‐related CRC were included in the survey. Additionally, 435 non‐T2DM CRC patients were meticulously matched in a 1:1 ratio, aligning with the T2DM cohort in terms of age group, year of diagnosis, and community management.

### 2.3 Cost Estimation

#### 2.3.1 Estimation of Direct Costs

Direct costs in this study encompass two primary categories: direct medical and direct nonmedical expenses. Direct medical costs include expenditures related to drug costs and outpatient hospitalization incurred in the preceding year. Direct nonmedical costs pertain to transportation, accommodation, and nutrition expenses arising during the course of medical treatment.

#### 2.3.2 Estimation of Indirect Costs

Indirect costs are defined as the economic impact of work absences due to disease diagnosis and treatment, or the inability to perform daily labor. This also includes costs associated with the assistance provided by relatives and friends. We employed the human capital method to calculate these costs, excluding the economic burden of premature mortality. The formula for calculating days of missed work incorporates the duration of hospital stays and outpatient visits. The indirect economic burden was then determined by multiplying the total missed workdays by the per capita national income (divided by 365 days) in Shanghai for the year 2022. The amount was further adjusted using a discount rate of 5% to account for the discounted value over time.

#### 2.3.3 Estimation of Intangible Costs

Intangible costs reflect the monetized loss of quality of life due to the psychological, mental, and daily life impacts of the disease on patients. This study utilized the quality‐adjusted life year (QALY) measurement method, commencing with an assessment of patient quality of life using the European Quality of Life Scale (EQ‐5D). The health utility value for CRC patients was calculated based on a scoring system established for the general Chinese population. The intangible economic burden was then computed by multiplying the lost healthy life years by the per capita national income and adjusted for productivity weights. These weights adhere to international standards, with specific values assigned for different age groups, and the per capita national income was based on the 2022 disposable income of residents at the sample location.

### 2.4 Statistical Analysis

The statistical analyses in this study were rigorously conducted using Stata V.15.1 software. To assess categorical variables, chi‐square tests were employed, whereas continuous variables were evaluated through the application of Student's *t*‐tests. Further, we utilized descriptive analysis techniques and multivariate logistic regression to explore the associations between demographic factors and the disease variables under study. The determination of statistical significance was grounded in two‐tailed *p* values, with *p* < 0.05 considered statistically significant.

Owing to the nonrandomized nature of participant selection in the study design, we implemented PSM to address potential imbalances between groups at baseline. These propensity scores were computed via logistic regression analysis, incorporating baseline characteristics as independent variables. Notably, variables that were statistically significant (*p* < 0.05) or deemed potential confounders were included in the final model. In an effort to mitigate the influence of confounding factors, cases of diabetic CRC were matched to nondiabetic controls using these propensity scores, adhering to a 1:1 ratio of controls to cases. The logistic regression model for estimating propensity scores included variables such as age, TNM stage, cancer type, hospitalization, surgery, chemotherapy, radiotherapy, and targeted immunotherapy. The normalization bias for the majority of the matched variables is below 10%.

To compare baseline characteristics between groups, we conducted Student's *t*‐test, chi‐square test, and Cochran–Mantel–Haenszel test. Specifically, Student's *t*‐test was applied for continuous variables such as age, the chi‐square test for categorical variables like sex, and the Cochran–Mantel–Haenszel test for nominal variables. Additionally, standardized differences were calculated to thoroughly examine and contrast the distributions of covariates between the case and control groups before and after the application of PSM.

## 3 Results

### 3.1 Baseline Characteristics

In this study, a total of 818 patients met the predefined inclusion criteria. Following the application of PSM, the analysis encompassed 752 participants. This cohort was balanced between 376 cases of diabetic CRC and 376 nondiabetic controls. The baseline characteristics of both cases and controls, pre‐PSM and post‐PSM, are systematically presented in Table 1.

**TABLE 1:** Baseline characteristics of subjects before and after PSM.

Baseline characteristics	Before PSM			After PSM		
	Treated group (*n* = 383)	Control group (*n* = 435)	*p*	Treated group (*n* = 376)	Control group (*n* = 376)	*p*
Sex			0.7			0.82
Male	227 (59.3)	252 (57.9)		223 (59.3)	220 (58.5)	
Female	156 (40.7)	183 (42.1)		153 (40.7)	156 (41.5)	
Age			< 0.01			0.09
< 65	24 (6.3)	65 (14.9)		20 (5.3)	32 (8.5)	
≥ 65	359 (93.7)	370 (85.1)		256 (94.7)	344 (91.5)	
Education level			< 0.01			< 0.01
Primary school (grade 1–6)	34 (8.9)	30 (6.9)		34 (9.0)	25 (6.7)	
Middle school (grade 7–9)	138 (36.0)	238 (54.7)		136 (36.2)	210 (55.9)	
High school (grade 10–12)	137 (35.8)	114 (26.2)		134 (35.6)	98 (26.1)	
College or higher (including associate degrees)	74 (19.3)	53 (12.2)		72 (19.2)	43 (11.4)	
Annual household income			0.58			0.68
Below 50,000 RMB	16 (4.2)	14 (3.2)		15 (4.0)	10 (2.7)	
50,000 to 100,000 RMB (including 100,000)	119 (31.1)	153 (35.2)		118 (31.4)	130 (34.6)	
100,000 to 150,000 RMB (including 150,000)	163 (42.6)	165 (37.9)		160 (42.6)	148 (39.4)	
150,000 to 200,000 RMB (including 200,000)	57 (14.9)	68 (15.6)		56 (14.9)	57 (15.2)	
Above 200,000 RMB	28 (7.3)	35 (8.1)		27 (7.2)	31 (8.2)	
Types of medical insurance			0.01			0.01
Urban Resident Basic Medical Insurance	133 (34.7)	196 (45.1)		133 (35.4)	174 (46.3)	
Urban Employee Basic Medical Insurance	241 (62.9)	233 (53.6)		234 (62.2)	196 (52.1)	
Else	9 (2.4)	6 (1.4)		9 (2.4)	6 (1.6)	
Hospitalization within 1 year after tumor diagnosis			< 0.01			< 0.01
Yes	324 (84.6)	308 (70.8)		317 (84.3)	285 (75.8)	
No	59 (15.4)	127 (29.2)		59 (15.7)	91 (24.2)	
Hospital Grade			0.32			0.34
Tertiary Hospital	289 (89.2)	282 (91.6)		283 (89.3)	261 (91.6)	
Secondary Hospital	35 (10.8)	26 (8.4)		34 (10.7)	24 (8.4)	
Cancer site			0.55			0.94
Colon cancer	237 (61.9)	278 (63.9)		234 (62.2)	233 (62.0)	
Rectal cancer	146 (38.1)	157 (36.1)		142 (37.8)	143 (38.0)	
TNM stage			0.2			0.16
0	38 (9.9)	43 (9.9)		37 (9.8)	29 (7.7)	
I	117 (30.5)	163 (37.5)		117 (31.1)	150 (39.9)	
II	110 (28.7)	112 (25.7)		110 (29.3)	101 (26.9)	
III	97 (25.3)	89 (20.5)		96 (25.5)	82 (21.8)	
IV	21 (5.5)	28 (6.4)		16 (4.3)	14 (3.7)	
Fistula surgery			0.06			0.05
Yes	35 (9.1)	25 (5.7)		35 (9.3)	21 (5.6)	
No	348 (90.9)	410 (94.3)		341 (90.7)	355 (94.4)	
Surgery or not			0.01			0.18
Yes	379 (99.0)	418 (96.1)		372 (98.9)	375 (99.7)	
No	4 (1.0)	17 (3.9)		4 (1.1)	1 (0.27)	
Radiotherapy			0.98			0.85
Yes	16 (4.2)	18 (4.1)		14 (3.7)	13 (3.5)	
No	367 (95.8)	417 (95.9)		362 (96.3)	363 (96.5)	
Chemotherapy			0.01			0.01
Yes	150 (39.2)	131 (30.1)		146 (38.8)	113 (30.1)	
No	233 (60.8)	304 (69.9)		230 (61.2)	263 (70.0)	
Targeted immunotherapy			0.76			0.56
Yes	11 (2.9)	11 (2.5)		7 (1.9)	5 (1.3)	
No	372 (97.1)	424 (97.5)		369 (98.1)	371 (98.7)	

Prior to the implementation of PSM, comparisons between the treated group and control group revealed similarities in variables such as gender, annual household income, and cancer site. However, notable differences were observed in aspects such as age, education level, and marital status, reaching statistical significance (*p* < 0.05). Subsequent to PSM, these characteristic disparities, particularly in age, the presence of surgical intervention, and the administration of chemotherapy, were significantly attenuated, illustrating no statistical differences (*p* > 0.05) between the intervention and control groups.

### 3.2 Costs

The comprehensive analysis of the economic burden of CRC, encompassing direct, indirect, and intangible costs, is detailed in Table 2. This table delineates the cost components per CRC patient, differentiated between diabetic and nondiabetic populations.

**TABLE 2:** Mean annual direct costs, indirect costs, and intangible costs per colorectal cancer patient in Shanghai, China (RMB).

Baseline characteristics	Diabetes colorectal cancer				Nondiabetic colorectal cancer			
	Direct costs		Indirect costs	Intangible costs	Direct costs		Indirect costs	Intangible costs
	Medical costs	Nonmedical costs			Medical costs	Nonmedical costs		
Sex								
Male	58,109.04	9749.05	269.69	3138.74	52,308.86	8215.78	233.67	2388.77
Female	55,530.13	8626.95	344.50	4276.46	44,174.41	7621.84	251.60	2974.91
Age								
< 65	50,531.95	6749.60	475.00	5282.52	64,288.09	6906.03	241.87	3507.57
≥ 65	57,426.37	9435.31	290.31	3507.27	47,505.64	8068.27	241.04	2550.51
Education level								
Primary school (grade 1–6)	41,938.97	8020.82	571.46	6416.33	31,529.60	3818.92	417.79	4306.58
Middle school (grade 7–9)	46,529.01	6653.91	250.51	2950.84	48,074.28	7808.47	244.54	2505.44
High school (grade 10–12)	62,020.49	10,315.04	258.05	3079.60	45,080.33	7023.90	187.60	2549.63
College or higher (including associate degrees)	74,858.47	12,973.69	344.06	4473.64	72,033.67	13,322.91	243.61	2463.84
Annual household income								
Below 50,000 RMB	69,036.87	12,566.87	700.28	8723.13	41,590.40	14,555.40	222.52	4251.17
50,000 to 100,000 RMB (including 100,000)	50,861.84	7036.55	356.63	4225.40	42,200.97	10,026.46	277.69	3092.18
100,000 to 150,000 RMB (including 150,000)	57,452.01	9661.09	226.92	2907.66	55,391.89	6173.10	221.16	2440.96
150,000 to 200,000 RMB (including 200,000)	62,469.00	12,146.79	330.88	3683.24	36,752.91	5482.02	258.92	2694.17
Above 200,000 RMB	63,947.85	9227.82	201.04	1974.33	71,103.61	10,367.45	156.22	977.15
Types of medical insurance								
Urban Resident Basic Medical Insurance	57,897.93	11,551.83	317.30	4492.94	49,035.52	9839.32	229.76	2274.38
Urban Employee Basic Medical Insurance	55,760.15	8088.51	275.64	3066.96	48,457.24	6193.71	251.48	2969.66
Else Hospital	78,458.56	7206.33	683.43	4334.32	61,559.50	11744.83	231.71	1970.35
Hospitalization within 1 year after tumor diagnosis								
Yes	67,340.26	10,168.02	278.78	3417.78	63,159.74	8382.41	251.43	2661.04
No	1823.12	4588.10	414.87	4589.85	4380.58	6675.75	208.81	2540.87
Hospital Grade								
Tertiary	64,588.74	6694.97	407.22	4261.95	67,751.79	3049.21	166.25	1802.50
Secondary Hospital	67,670.83	10,585.28	263.35	3316.36	62,737.48	8872.82	259.26	2739.99
Cancer site								
Colon cancer	53,970.56	9857.46	297.72	3475.59	49,418.42	7741.88	216.35	2402.34
Rectal cancer	62,150.11	8361.38	304.10	3809.51	48,144.51	8340.00	281.45	3006.09
TNM stage								
0	50,020.16	6047.11	128.11	1630.07	45,272.31	4667.79	97.24	1276.51
I	40,970.72	5639.88	335.87	3715.47	34,159.03	6565.85	278.39	3074.96
II	74,008.39	14,073.89	248.69	3319.30	57,368.84	8211.29	198.19	2223.48
III	61,272.68	8570.63	370.70	4162.11	66,004.56	9904.84	266.94	2584.41
IV	49,187.94	14,965.25	366.89	5908.06	53,984.29	16,764.14	298.14	3918.52
Fistula surgery								
Yes	75,467.97	8714.00	320.48	3293.58	93,761.24	10,534.38	347.68	3187.05
No	55,170.23	9351.82	298.04	3633.32	46,282.17	7817.63	234.81	2599.12
Surgery or not								
Yes	57,658.81	9305.67	294.11	3477.74	49,061.52	7975.37	241.46	2636.51
No	1337.00	8063.00	860.10	15,129.88	1089.00	5715.00	109.71	923.48
Radiotherapy								
Yes	88,980.86	13,132.14	484.32	2917.14	127,814.40	16,365.69	211.41	1498.51
No	55,825.12	9143.95	293.01	3628.17	46,109.01	7668.66	242.18	2672.55
Chemotherapy								
Yes	73,507.89	12,272.11	259.91	3426.99	74561.12	9707.00	206.08	2259.59
No	46,618.59	7401.01	325.67	3712.60	37,923.01	7222.77	256.16	2791.95
Targeted immunotherapy								
Yes	57,350.94	10,822.29	491.48	8645.65	115,587.00	12,654.8	112.70	635.29
No	41,704.29	9263.43	296.50	3506.01	48,035.64	7906.21	242.84	2658.87
Mean costs	57,059.65*	9292.45	300.13	3601.70	48,933.93	7969.35	241.11	2631.96
Total costs	70,253.93*				59,776.36			

* *p* < 0.05.

Among patients with diabetes‐associated CRC, the mean unit costs were estimated as follows: direct medical costs at ¥57,059.65, direct nonmedical costs at ¥9292.45, indirect costs at ¥300.13, and intangible costs at ¥70,253.93. In contrast, for the nondiabetic CRC cohort, these costs were calculated at ¥48,933.93, ¥7969.35, ¥241.11, and ¥2631.96 for direct medical, direct nonmedical, indirect, and intangible costs, respectively, culminating in a total estimated illness cost of ¥59,776.36.

Further, Table 2 provides a nuanced comparison of CRC costs across various demographics, including sex, age, education level, and TNM stage, within the surveyed communities. Notably, male patients incurred higher direct economic costs compared to females. Patients aged 65 years and above exhibited higher direct costs but lower indirect and intangible costs. An interesting trend observed was the fluctuation of CRC costs with annual household income, initially decreasing and then increasing. Patients enrolled in basic medical insurance for urban employees demonstrated lower disease costs. Additionally, hospitalized patients and those undergoing surgical procedures faced heightened illness costs.

Focusing on type 2 diabetes mellitus (T2DM) CRC patients, specific cost analyses revealed that direct medical costs for rectal cancer amounted to ¥62,150.11. In Stage II diagnosed patients, direct medical and nonmedical costs were estimated at ¥74,008.39 and ¥14,073.89, respectively. Surgical T2DM patients incurred direct medical costs of ¥57,658.81, contrasting with ¥1337.00 for nonsurgical patients. The intangible economic burdens for surgical and nonsurgical T2DM patients were calculated at ¥3477.74 and ¥15,129.88, respectively. In the non‐T2DM CRC group, rectal cancer patients faced direct nonmedical costs of ¥48,144.51. For Stage III patients, direct medical and nonmedical costs were ¥66,004.56 and ¥9904.84, respectively. Surgical non‐T2DM patients bore direct medical costs of ¥49,061.52, compared to ¥1089.00 for nonsurgical patients, with intangible burdens at ¥2636.51 and ¥923.48, respectively.

## 4 Discussion

The ever‐increasing costs of health systems around the world, especially in developing countries such as China, have become a major concern of health managers and policymakers, the main reason for which is the growing number of noncommunicable, chronic, and incurable diseases such as cancer. The significant advancement of diagnostic and therapeutic technologies has made the cost of CRC treatment to be remarkably high. The aim of the present study was to determine whether there was a difference in the cost of disease between diabetic and nondiabetic CRC patients based on the analysis of the cost level of disease in CRC patients and determine the economic burden of CRC in patients who had been referred to the Hongkou District Tumor Report Management System to Shanghai, China.

The findings of this study demonstrate a significant economic burden of CRC on healthcare systems. The average total cost per patient in this study encompasses direct medical costs, direct nonmedical costs, indirect costs, and intangible costs, with the highest proportion attributed to direct medical costs. The results of this study indicate that the overall economic burden of CRC patients accounts for approximately 0.103% of the gross domestic product (GDP) in Hongkou District, Shanghai, China. This finding is consistent with other studies on the economic burden of CRC in China, which reported an overall economic burden of approximately 17.05 billion RMB, representing about 0.189% of the national GDP, with 91.4% of expenses classified as direct medical costs [13]. This is consistent with findings from studies on the economic burden of CRC in Europe [14]. Irrespective of diabetes status, higher educational attainment correlates with increased expenses among CRC patients who underwent hospitalization, surgery, radiotherapy, chemotherapy, or targeted immune therapy, resulting in a heavier economic burden [15, 16]. Notably, this study identifies surgical costs as the highest component of average direct medical expenses per patient. The elevated expenses associated with surgery primarily stem from the imperative need for organ resection as a crucial aspect of initiating treatment, often conducted before chemotherapy, to prevent metastasis and impede disease progression to other organs like the liver and lungs [17]. This aligns with the outcomes observed in this research.

Comparative analysis of costs between T2DM and non‐T2DM CRC patients reveals a significantly higher economic burden among patients with T2DM. Direct medical costs, direct nonmedical costs, indirect costs, and intangible burdens are all higher in T2DM patients compared to nondiabetic individuals, with direct medical costs notably elevated. Noteworthy is the observation that among patients diagnosed at Stage II with concomitant diabetes, direct medical costs are highest compared to all other stages, significantly surpassing those without diabetes in the same stage. This can be attributed to the predominant use of surgical resection in Stage II CRC cases, where most patients require surgical intervention in the early stages of cancer. In contrast, the majority of Stage III and IV cases, occurring at later TNM stages, tend toward conservative treatments like radiotherapy [18]. Therefore, Stage II patients accrue higher direct medical costs due to surgical interventions. Moreover, a study assessing healthcare‐associated infections among Chinese CRC surgical patients discovered an infection incidence rate of 8.93% [19], exceeding the national incidence rate in American hospitals (4.0%) [20]. This resulted in an additional medical expense of $1589.30 per patient, potentially leading to postoperative infections and complications, significantly amplifying the direct economic burden on CRC surgical patients in China. The presence of Type 2 diabetes elevates the probability and severity of postoperative infections and complications. A study on the disease burden of diabetes indicated that 76.4% of diagnosed patients reported at least one complication. The treatment costs for patients with complications exhibit a linear relationship, with costs for patients with complications being 3.36 times higher than those without complications [21]. This accounts for the higher economic burden among surgical patients with diabetes in this study compared to nondiabetic surgical patients. Complications also lead to increased nonmedical expenses such as transportation, accommodation, and a decline in quality of life, thereby generating higher indirect and intangible economic burdens. This elucidates the higher direct nonmedical costs, indirect costs, and intangible burdens in patients with Type 2 diabetes.

CRC stands as one of the cancers that contribute significantly to increased economic costs. This study identifies CRC as imposing substantial economic burdens on patients, with patients having Type 2 diabetes experiencing an even heavier burden. To alleviate the socioeconomic burden of CRC, emphasis should be placed on early treatment and examinations, alongside lifestyle modifications. In considering the socioeconomic burden of CRC, increasing CRC screening rates may help alleviate the economic burden. Additionally, the rapid aging population and increased life expectancy have raised relative cancer survival rates. However, due to the escalation in chronic disease prevalence, patient quality of life decreases, and economic burdens rise. Therefore, there is a need to enhance preventive measures and training regarding risk factors for CRC and other chronic diseases, focusing on primary and secondary disease prevention, while timely adjustments in healthcare policies are imperative.

Several limitations exist in this study. Firstly, the utilization of the human capital approach to estimate productivity loss relies on estimating financial market wage disparities. The friction cost method estimates employer expenses rather than patient expenses. Hence, the human capital approach may identify larger cost differences than the friction cost method, potentially resulting in an overestimation of productivity loss. Secondly, the estimation of transportation costs, caregiver expenses, and the time loss of caregivers and patients might lead to the overestimation of certain costs. Thirdly, future research should consider the costs of complementary and alternative medicine. Fourthly, the inability to estimate the psychological distress experienced by patients and their families, as well as losses incurred due to self‐disinfection tools and medications required after CRC treatment/surgery, represents a limitation. Finally, despite PSM, some residual confounding variables persist such as comorbidities, which could influence both the burden of disease and patient outcomes, potentially leading to biased results if not properly accounted for, contributing to uncertainty in conclusions. Future studies should aim to integrate these critical factors to provide a more comprehensive understanding of disease burden in this population. And future studies should further investigate these imbalances or seek larger sample sizes to enhance the reliability of research findings.

To mitigate the economic burden of CRC, it is imperative to promote primary and secondary prevention while focusing on the detrimental effects of chronic diseases such as diabetes [22, 23]. Increasing participation rates in CRC screening might be cost‐effective. Future research should concentrate on addressing the issue in depth [24].

## 5 Conclusion

This study reveals the substantial economic burden of CRC on healthcare systems, particularly exacerbated in patients with T2DM. Direct medical costs, especially from surgical interventions, are significantly higher in diabetic patients due to increased complications and infections. To alleviate this burden, early diagnosis, increased CRC screening, and lifestyle modifications are essential. Emphasizing preventive measures and adjusting healthcare policies to address CRC and chronic disease risk factors can help reduce these economic impacts. Future research should further explore the economic implications and address the study's limitations to develop more effective mitigation strategies.

## Author Contributions

**Zhi‐xu Zhu:** software (equal), visualization (lead), writing – original draft (lead). **Yu‐xin Peng:** software (equal), writing – original draft (equal), writing – review and editing (equal). **Jing‐hong Ye:** data curation (equal), funding acquisition (equal), investigation (equal), project administration (equal), resources (equal), supervision (equal), writing – review and editing (equal). **Yan Wei:** conceptualization (equal), funding acquisition (equal), project administration (equal), resources (equal), supervision (equal), writing – review and editing (equal).

## Ethics Statement

The study was approved by the Medical Research Ethics Committee of the School of Public Health, Fudan University [IRB No. 2024‐06‐1129] as a review exemption.

## Consent

Written informed consent was obtained from all participants prior to their inclusion in the cohort study.

## Conflicts of Interest

The authors declare no conflicts of interest.

## Data Availability

The datasets used and analyzed during this study are available upon reasonable request. The corresponding author should be contacted for the request to access the raw data.
